# Increased heat shock protein 70 (Hsp70) serum levels and low NK cell counts after radiotherapy – potential markers for predicting breast cancer recurrence?

**DOI:** 10.1186/s13014-019-1286-0

**Published:** 2019-05-10

**Authors:** Anna Rothammer, Eva K. Sage, Caroline Werner, Stephanie E. Combs, Gabriele Multhoff

**Affiliations:** 1 Center for Translational Cancer research (TranslaTUM), Radiation Immuno-Oncology Group, Technical University of Munich (TUM), School of Medicine, Klinikum rechts der Isar, Einsteinstr. 25, 81675 Munich, Germany; 2 Department of Radiation Oncology, Technical University of Munich (TUM), School of Medicine, Klinikum rechts der Isar TUM, Munich, Germany; 3 Deutsches Konsortium für Translationale Krebsforschung (DKTK), partner site, Munich, Germany; 4 Institute of Radiation Medicine (IRM), Helmholtz Zentrum München, Oberschleißheim, Germany

**Keywords:** Breast carcinoma prediction, DAMPs, Free Hsp70, Immunophenotyping, Radiotherapy

## Abstract

**Background:**

Breast cancer is the most common invasive tumor in women worldwide and the second cause of cancer-related deaths. After breast conserving surgery the tumor bed gets irradiated. Radiation-induced tumor cell death has been found to be associated with the release of damage-associated molecular patterns (DAMPs) including free Hsp70 that can stimulate inflammatory immune responses. Therefore, Hsp70 serum levels as well as the composition of lymphocyte subpopulations have been measured in breast cancer patients during therapy and in the follow-up period as potential predictors for clinical outcome.

**Methods:**

The serum of 40 breast cancer patients, who received a breast-conserving surgery and adjuvant radiotherapy (RT) was examined for soluble, free Hsp70 using the R&D Human HSP70 DuoSet and lipHsp70 ELISA. Lymphocyte subpopulations and total lymphocyte counts were analysed by multiparameter flow cytometry in the peripheral blood. Blood samples were collected before (t1), after 30 Gy (t2) and 60 Gy (t3), 6 weeks (t4), 6 months (t5) and 1 year (t6) after RT. Clinical responses were assessed regularly up to 5 years after RT.

**Results:**

Patients who developed a contralateral recurrence or metastases within the first 2 years after RT had significantly higher serum Hsp70 values at the end of RT (t3; *p* = 0.03) up to 6 weeks after RT (t4; *p* = 0.007) compared to patients who either remained disease-free or developed a secondary endometrial carcinoma. Clinicopathological parameters such as age, tumor size, grading and TNM-stage of the resected tumors, adjuvant chemotherapy and irradiation dose did not affect serum Hsp70 levels. Elevated free Hsp70 levels might be indicative for a chronic inflammatory response which could support tumor recurrence. Lymphocyte subpopulation analysis revealed lower NK cell counts after RT in recurrence/metastases patients as compared to disease-free patients. In contrast, no significant changes were observed in the proportion of T and B cells.

**Conclusion:**

Longitudinal elevated serum levels of free Hsp70 up to 6 weeks after RT and dropping NK cell counts might be predictive for an unfavourable prognosis in patients with breast cancer.

## Background

With 2,088,849 newly diagnosed cases in 2018, reflecting 25.4% of all tumor entities, breast cancer is the most common invasive cancer in women worldwide (World Cancer Research Fund). In early tumor stages, a breast-conserving surgery with adjuvant RT is the standard of care. A comparison of accelerated partial breast irradiation using multicatheter brachytherapy and whole breast irradiation after breast conserving surgery revealed no significant differences in the 5 year local control, disease-free and overall survival [1]. Postoperative RT of the tumor bed results in lower rates of local recurrence [2] because remaining viable tumor (stem) cells might be depleted [3]. Irradiation- as well as chemotherapy-induced stress can result in the release of damage-associated molecular patterns (DAMPs) by tumor cells which induce immunogenic cell death [4–7]. These DAMPs including calreticulin, extracellular ATP, high mobility group box 1 (HMGB1), HSPs derived from programmed, caspase-independent cell death termed necroptosis [8] are known to trigger acute inflammation and thereby might elicit protective antitumor immunity [9]. In contrast to acute, chronic inflammation has been shown to promote tumor growth [10]. Therefore, the impact of DAMPs in the context of acute and chronic inflammation need further investigations. The highly conserved member of the 70 kDa heat shock protein family Hsp70 is considered as a DAMP when released as a free molecule by dying cancer cells [11] whereas exosomal Hsp70 (exHsp70) which is actively released by viable tumor cells serves as a marker for viable tumor mass [12, 13]. Elevated exHsp70 serum levels as detected by the lipHsp70 ELISA [14] that detects both, free and exosomal Hsp70 have been found in breast cancer patients [15], patients with hepatocellular carcinoma, lung cancer [16], colorectal carcinoma [17, 18] and other cancer entities [19]. Although high exHsp70 levels are generally associated with an adverse prognosis also in breast cancer patients [20, 21], less is known about the role of free Hsp70 as a predictive marker for therapy response. Therefore, we investigated serum levels of free Hsp70 as well as the immunophenotype in breast cancer patients before, during, after RT and in the follow-up period up to 1 year to study potential immunomodulatory effects of circulating, free Hsp70.

## Methods

### Study collective

**Fig. 1:**
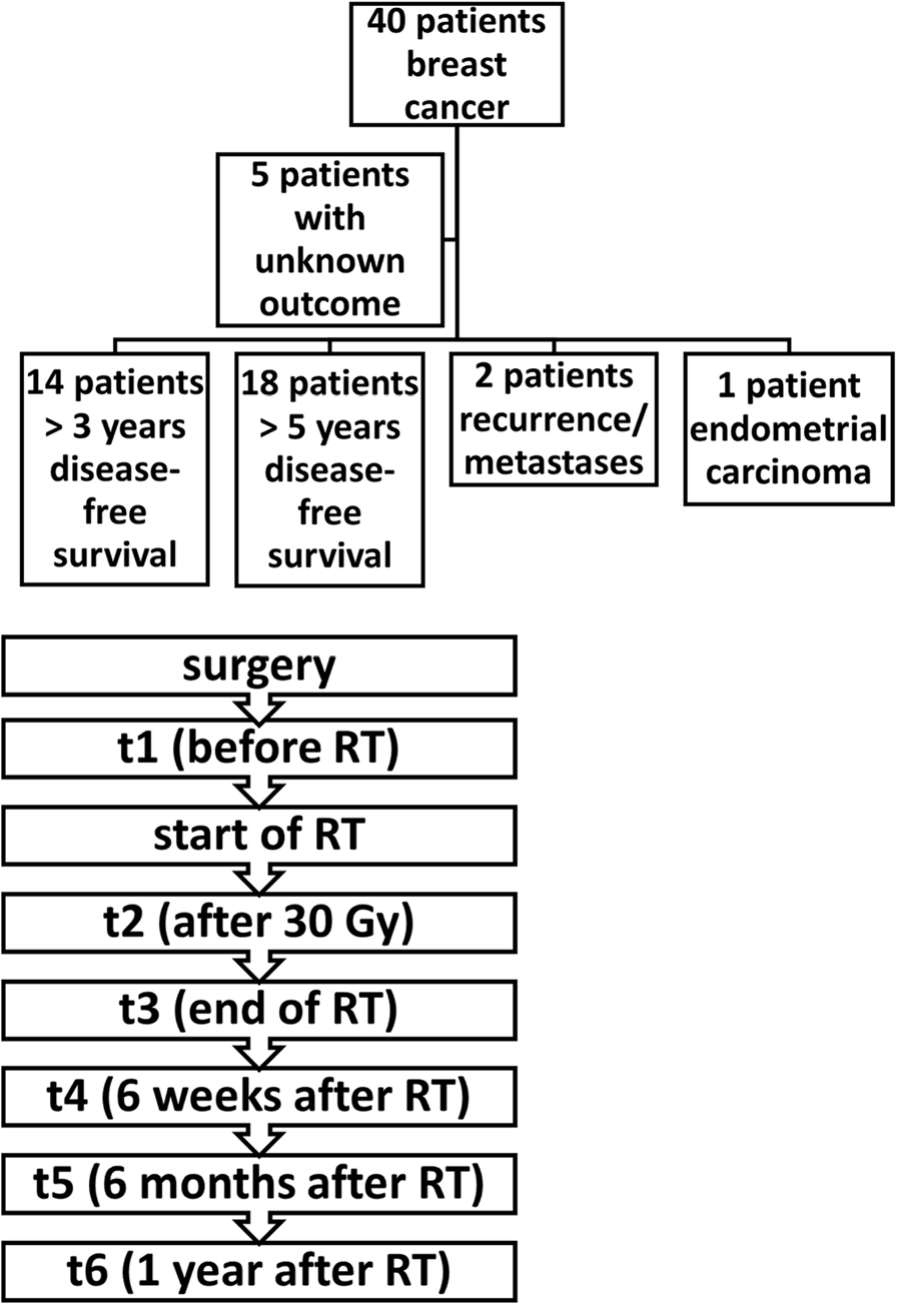
Patient evaluation. Schematic illustration of the patient cohort, therapy and clinical outcome. Forty patients with breast cancer were included into the study, 5 patients had an unknown outcome, 14 patients remained disease-free for at least 3 years after RT and 18 patients remained disease-free for at least 5 years after RT, 2 patients developed contralateral recurrence or distant metastasis, 1 patient developed a secondary endometrial carcinoma after 2 years. The time-points of blood withdrawals (t1, before RT; t2, after 30 Gy; t3, end of RT; t4, 6 weeks after RT; t5, 6 months after RT; t6, 1 year after RT) in the course of therapy are indicated in the lower part of the graph

All patients (*n* = 40) included in the study, were females with unilateral invasive breast cancer receiving breast conserving surgery at the Department of Radiation Oncology, Klinikum rechts der Isar, Technische Universität München (TUM). Excluded were patients who had distant metastases, secondary tumors, neoadjuvant chemotherapy, mastectomy or a previous RT. Five patients could not be analysed because no follow-up data were available. After surgery, the patients received a tangential irradiation of the breast. The median total dose was 60 Gy, including the sequential boost, fractioned in daily 2 Gy irradiations. Nine patients received an adjuvant chemotherapy (5-floururacil, epirubicin, cyclophosphamide, FEC) after surgery and two to 6 weeks before start of RT. At six different time points, blood samples (serum and EDTA blood) were collected: after surgery and before RT (t1), after 30 Gy (t2), at the end of RT (t3), 6 weeks (t4), 6 months (t5) and 1 year after RT (Fig. 1). The patients’ clinical outcome was investigated regularly up to 4 years after the last blood sample was taken and up to 5 years after the end of RT. The trial was approved by the local ethical committee of the Medical Faculty, Klinikum rechts der Isar, TU München. All patients gave written informed consent before start of the study.

### Hsp70 ELISA

The serum of patients was investigated for free Hsp70 using a modified protocol of the R&D Human HSP70 DuoSet ELISA kit. A 96-well plate was coated with the capture antibody 24 h before starting the test. After washing and blocking with 1% bovine serum albumin (BSA) in phosphate buffered saline (PBS) for 2 h, the wells were incubated with 50 μl serum diluted 1:5 with 0.5% Triton, 1 mM EDTA in PBS and freshly prepared, titrated protein standard for 2 h. After an incubation period of 2 h with the detection antibody, streptavidin conjugated horseradish-peroxidase (R&D systems) was added for 20 min and freshly prepared H_2_O_2_ and tetramethylbenzidine was added for another 20 min. The colour reaction was stopped by adding 2 N H_2_SO_4_ and immediately thereafter, the optical density was determined at 450 nm and 570 nm on an ELISA reader (Victor Multilable Plate Reader; PerkinElmer, Germany). The concentration of free Hsp70 in the serum was calculated based on the differences in the optical densities at both wavelengths in relation to the protein standard. The lipHsp70 ELISA was performed as described previously [14], and median values were determined.

### Flow cytometry

Lymphocyte subpopulations were immunophenotyped by multiparameter flow cytometry on a FACSCalibur flow cytometer (BD Biosciences) according to a protocol described previously [22]. Briefly, 100 μl EDTA-blood was mixed with the fluorescence-labelled antibody combinations directed against CD3^+^ T cells, CD4/CD25/FoxP3^+^ regulatory T cells, CD56^+^ NK cells and CD19^+^ B cells for 15 min in the dark. After washing in PBS/10% heat-inactivated fetal calf serum (Sigma F7524) and erythrocyte lysis (BD, 349202) in ammonium chloride (NH_4_Cl, 8.26 g in ddH_2_O), potassium bicarbonate (KHCO_3_, 1 g), EDTA (0.037 g) buffer (pH 7.3), 50,000 to 100,000, CD45^+^ cells were analysed. The CD4^+^CD25^+^ T cell population was stained with the FoxP3-PE antibody after fixation (BD51–9005451) and permeabilization (BD51–9005450) to determine regulatory T cell counts. The respective percentage of lymphocyte subpopulations is defined as the proportion of cells stained positively for a specific antibody within the lymphocyte gate. Isotype-matched, fluorescence-labelled antibodies are used as negative controls.

### Statistics

Each sample is measured four times in at least two independent experiments. For the following evaluations, the mean values of at least four measurements are used. Differences between two data sets are calculated using the Mann-Whitney-U rank sum test when the data were not normally distributed. The Gaussian distribution of the data was tested with the Normality test. When more than two data sets were compared the Kruskal-Wallis-test was applied. Differences in *p*-values < 0.05 were considered as statistically significant. The software used was IBM SPSS Statistics. Only patients (*n* = 35) with proven outcome were analysed.

## Results

The patients’ characteristics and therapies are summarized in Table 1. The age of the 35 analysed patients at diagnosis ranged from 43 to 83 years and the size of the resected tumors ranged between 0.2 and 2.2 cm. With respect to TNM, 30 patients (86%) were in stage T1/N0 and 5 patients (14%) in T2/N1; none of the patients had distant metastases (M0). Seven patients were diagnosed with grade 1, 26 with grade 2, and 2 with grade 3 tumors. Eight patients received additional chemotherapy after surgery and RT. All patients were estrogen (ER^+^) and progesterone receptor (PR^+^) positive and apart form 1 patient who refused, 34 patients were treated with antihormonal therapy, 12 of them started the therapy before (34%) and 22 (63%) after RT. Concerning RT, 3 patients received a total dose of only 40 Gy, 27 patients were irradiated with a total dose of 60 Gy, 25 of them received 50 Gy and a boost of the primary tumor region of 10 Gy, and five patients received a total dose of 66 Gy including a boost of 16 Gy.

**Table 1:** Patient’s characteristics, therapies and clinical outcome

Age (years)	N	%
40–49	8	23
50–59	11	32
60–69	11	31
> 70	5	14
Chemotherapy		
Yes	8	23
No	27	77
Hormone therapy		
Start before RT	12	34
Start after RT	22	63
No	1	3
Tumor size (cm)		
< 1	12	34
1–2	20	57
> 2	3	9
pTNM		
T1 (a-c)	30	86
T2	5	14
N0	30	86
N1	5	14
M0	35	100
G1	7	20
G2	26	74
G3	2	6
Hormone receptor status		
Estrogen receptor^+^	35	100
Progesteron receptor^+^	35	100
Total dose (Gy)		
40	3	9
60	27	77
66	5	14
Total dose of boost (Gy)		
10	25	72
16	5	14
Clinical outcome		
Disease-free > 3 years	14	40
Disease-free > 5 years	18	52
Endometrial cancer	1	3
Recurrence/metastases	2	6

The response assessment included clinical examination, mammography and sonography. Three and 5 years after the end of RT, 32 patients (14 and 18 patients, respectively) were disease-free. One patient developed an endometrial carcinoma and two patients showed a contralateral recurrence or developed distant metastases 2 years after RT (Fig. 1; Table 1). The clinical outcome of five patients remained unknown (Fig. 1), and therefore these patients were not considered in the analysis.

To study whether circulating Hsp70 levels were impacted by the age of the patient, Hsp70 serum levels were comparatively studied at the indicated time-points (before RT, after 30 Gy, end of RT, 6 weeks, 6 months and 1 year after RT) in the different age groups. As summarized in Table 2, no significant differences in the Hsp70 serum levels were detected in patients aged 40–49 (*n* = 8), 50–59 (*n* = 11), 60–69 (n = 11) and over 70 (*n* = 5) years at the different time-points of blood withdrawal. Regarding these results, we assumed that serum levels of free Hsp70 are age independent.

**Table 2:** Hsp70 serum levels (ng/ml) in different age groups of the breast cancer patients (mean ± standard deviation)

Age	N	Before RT	After 30 Gy	End of RT	6 weeks after RT	6 months after RT	1 yearafter RT
40–49	8	2.11 ± 0.78	2.47 ± 2.05	1.97 ± 0.64	1.77 ± 0.78	2.40 ± 1.31	2.33 ± 0.67
50–59	11	2.56 ± 0.99	2.11 ± 0.71	2.17 ± 0.51	2.55 ± 0.74	2.33 ± 1.03	2.07 ± 0.03
60–69	11	2.42 ± 0.76	2.09 ± 0.58	2.09 ± 0.40	2.15 ± 0.57	2.26 ± 0.67	2.48 ± 1.25
> 70	5	2.51 ± 1.62	2.14 ± 0.95	1.77 ± 0.54	2.43 ± 0.88	2.36 ± 1.18	2.29 ± 1.09
p		0.776	0.883	0.646	0.179	0.993	0.991

Kruskal-Wallis-test

A potential correlation of free Hsp70 serum levels with respect to the tumor size before resection, TNM stage and grading was also analysed at the different time-points. As shown in Table 3, the original tumor size, TNM stage or grading also did not influence the Hsp70 serum levels significantly. Since patients with distant metastases were not included into the study, this parameter was not analysed.

**Table 3:** Hsp70 serum levels (ng/ml) in relation to tumor size, TNM stages and grading of the previously resected tumors (mean ± standard deviation)

	N	Before RT	After 30 Gy	End of RT	6 weeks after RT	6 months after RT	1 year after RT
Tumor size (cm)							
< 1	12	2.62 ± 1.35	2.55 ± 1.69	2.11 ± 0.50	2.18 ± 0.78	2.71 ± 1.26	2.94 ± 1.26
1–2	20	2.26 ± 0.55	2.00 ± 0.60	2.04 ± 0.49	2.26 ± 0.75	2.17 ± 0.76	2.16 ± 0.58
> 2	3	2.56 ± 1.51	2.02 ± 0.96	1.80 ± 0.84	2.13 ± 0.88	1.79 ± 0.65	1.65 ± 0.80
p		0.990	0.833	0.809	0.976	0.379	0.132
Kruskal-Wallis-test							
pTNM							
T1 (a-c)	30	2.40 ± 0.95	2.19 ± 1.15	2.07 ± 0.49	2.19 ± 0.75	2.33 ± 0.97	2.50 ± 0.99
T2	5	2.43 ± 1.15	2.18 ± 1.01	1.85 ± 0.66	2.37 ± 0.78	2.29 ± 1.14	1.74 ± 0.68
p-value		1.00	0.982	0.536	0.671	0.962	0.162
Mann-Whitney-U-test							
N0	30	2.36 ± 0.98	2.17 ± 1.18	2.02 ± 0.50	2.18 ± 0.73	2.39 ± 1.05	2.32 ± 0.98
N1	5	2.70 ± 0.84	2.32 ± 0.73	2.16 ± 0.63	2.42 ± 0.94	2.00 ± 0.13	3.23
p		0.369	0.395	0.732	0.671	0.539	0.261
Mann-Whitney-U-test							
Grading							
G1	7	2.57 ± 1.52	2.13 ± 0.76	2.11 ± 0.67	2.22 ± 1.06	2.14 ± 0.49	2.56 ± 0.70
G2	26	2.38 ± 0.83	2.24 ± 1.26	2.02 ± 0.50	2.17 ± 0.70	2.35 ± 1.12	2.07 ± 0.66
G3	2	2.48 ± 0.72	1.96 ± 0.71	2.10 ± 0.03	2.39 ± 0.12	2.19 ± 0.33	2.91
p		0.849	0.985	0.993	0.781	0.776	0.239
Kruskal-Wallis-test							

To exclude chemotherapy as a potential factor that might impact serum Hsp70 levels, patients who received adjuvant chemotherapy (*n* = 27) were compared to those, who remained chemotherapy-free (*n* = 8; Table 4). Unexpectedly, the mean Hsp70 serum concentrations of patients with chemotherapy was slightly lower than that of patients without additional chemotherapy at 30 Gy and after end of RT, however, the differences failed to show statistical significance (*p* = 0.061; *p* = 0.113).

**Table 4:** Hsp70 serum levels (ng/ml) in patients with and without adjuvant chemotherapy (mean ± standard deviation)

Chemo-therapy	N	Before RT	After 30 Gy	End of RT	6 weeks after RT	6 months after RT	1 year after RT
No	27	2.52 ± 1.01	2.31 ± 1.18	2.12 ± 0.44	2.20 ± 0.66	2.36 ± 1.00	2.45 ± 0.95
Yes	8	2.02 ± 0.67	1.78 ± 0.81	1.79 ± 0.69	2.30 ± 1.08	2.21 ± 0.95	1.44 ± 0.81
p		0.236	0.061	0.113	0.934	0.771	0.198

Mann-Whitney-U-test

Similar to the findings obtained for chemotherapy, also differences in the total irradiation dose ranging from 40 Gy to 66 Gy, as well as differences in the boost dose (10 Gy versus 16 Gy) did not alter the Hsp70 serum levels significantly, at any of the tested time-points (Table 5).

**Table 5:** Hsp70 serum levels (ng/ml) in relation to different total and boost irradiation doses (mean ± standard deviation)

	N	Before RT	After 30 Gy	End of RT	6 weeks after RT	6 months after RT	1 year after RT
Total dose (Gy)							
40	3	2.24 ± 0.67	2.05 ± 0.36	1.88 ± 0.22	2.10 ± 0.45	2.19 ± 0.29	2.67 ± 0.71
60	27	2.42 ± 0.95	2.22 ± 1.21	2.04 ± 0.55	2.27 ± 0.82	2.18 ± 0.77	2.33 ± 1.07
66	5	2.41 ± 1.30	2.10 ± 1.02	2.13 ± 0.47	1.99 ± 0.28	3.42 ± 1.91	2.24 ± 0.72
p		0.947	0.838	0.719	0.921	0.629	0.597
Kruskal-Wallis-test							
Boost dose (Gy)							
0	5	3.20 ± 1.80	2.31 ± 0.47	2.14 ± 0.26	2.72 ± 0.55	2.62 ± 0.69	2.79 ± 0.61
10	25	2.30 ± 0.80	2.20 ± 1.25	2.02 ± 0.56	2.21 ± 0.82	2.12 ± 0.76	2.30 ± 1.13
16	5	2.41 ± 1.30	2.10 ± 1.02	2.13 ± 0.47	1.99 ± 0.28	3.42 ± 1.91	2.24 ± 0.72
p		0.638	0.598	0.853	0.480	0.429	0.351
Kruskal-Wallis-test							

**Fig. 2:**
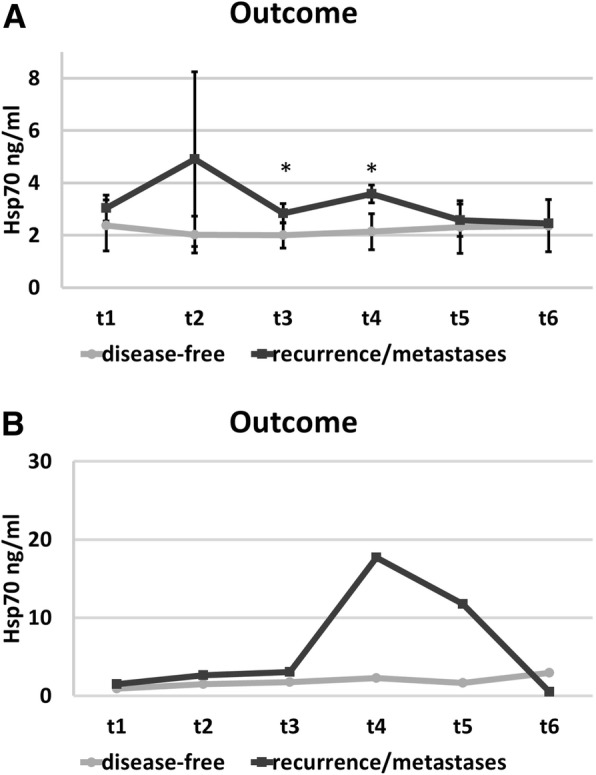
Kinetics of free Hsp70 serum concentrations before RT (t1), after 30 Gy during RT (t2), at the end of RT (t3), and in the follow-up period (t4; 6 weeks after RT; t5, 6 months after RT; t6, 1 year after RT) in patients who remained disease-free (closed circles) or patients who developed recurrence or distant metastases (closed squares), as determined by the R&D (**a**) and lipHsp70 ELISA (**b**). The values between the two groups differed significantly at t3 (*p* = 0.03) and t4 (*p* = 0.007) using R&D ELISA

However, a comparison of the free Hsp70 serum levels with the outcome revealed significant differences. Although the number of patients who showed recurrence was low both breast cancer patients who developed distant metastases or contralateral recurrence within the first 2 years after RT exhibited significantly higher median serum Hsp70 levels directly after RT (t3; *p* = 0.03) which remained significantly upregulated up to 6 weeks after RT (t4; *p* = 0.007) compared to the group of patients who remained disease-free for 3 and 5 years, respectively (Fig. 2a). The Hsp70 serum levels of the patient who was diagnosed with a secondary endometrium carcinoma 2 years after RT ranged within that of healthy individuals (2.74 + 1.07 ng/ml, *n* = 131 healthy donors) throughout the course of disease (Table 6). Blood samples of patients with other tumor entities such as squamous cell carcinoma of the head and neck (SCCHN), lung cancer, or glioblastoma at diagnosis revealed higher Hsp70 serum levels, as determined with the R&D ELISA compared to those of healthy controls (Table 6).

**Table 6:** Hsp70 serum levels in healthy human individuals and patients with different tumor entities (mean ± standard deviation) as determined by the R&D ELISA assay. *Abbreviations*: *SCCHN* squamous cell carcinoma of the head and neck

Healthy controls vs tumor patients	Number	Hsp70 serum levels (ng/ml)
Healthy controls	131	2.74 ± 1.07
SSCHN	22	3.51 ± 3.58
Lung cancer	168	5.40 ± 4.56
Glioblastoma	141	14.1 ± 10.63

A measurement of the serum samples of the breast cancer patients at the different time-points after surgery using the lipHsp70 ELISA revealed similar results to those assessed by the R&D ELISA. However, due to the higher sensitivity of the lipHsp70 ELISA, the values were higher than that measured with the R&D ELISA (Fig. 2b). Since all patients were tumor-free at the time-point of blood collection, we assume that the lipHsp70 ELISA only detects free but not exosomal Hsp70. Due to the limited amount of serum samples of the patients the lipHsp70 ELISA could be performed only once in triplicates.

**Fig. 3:**
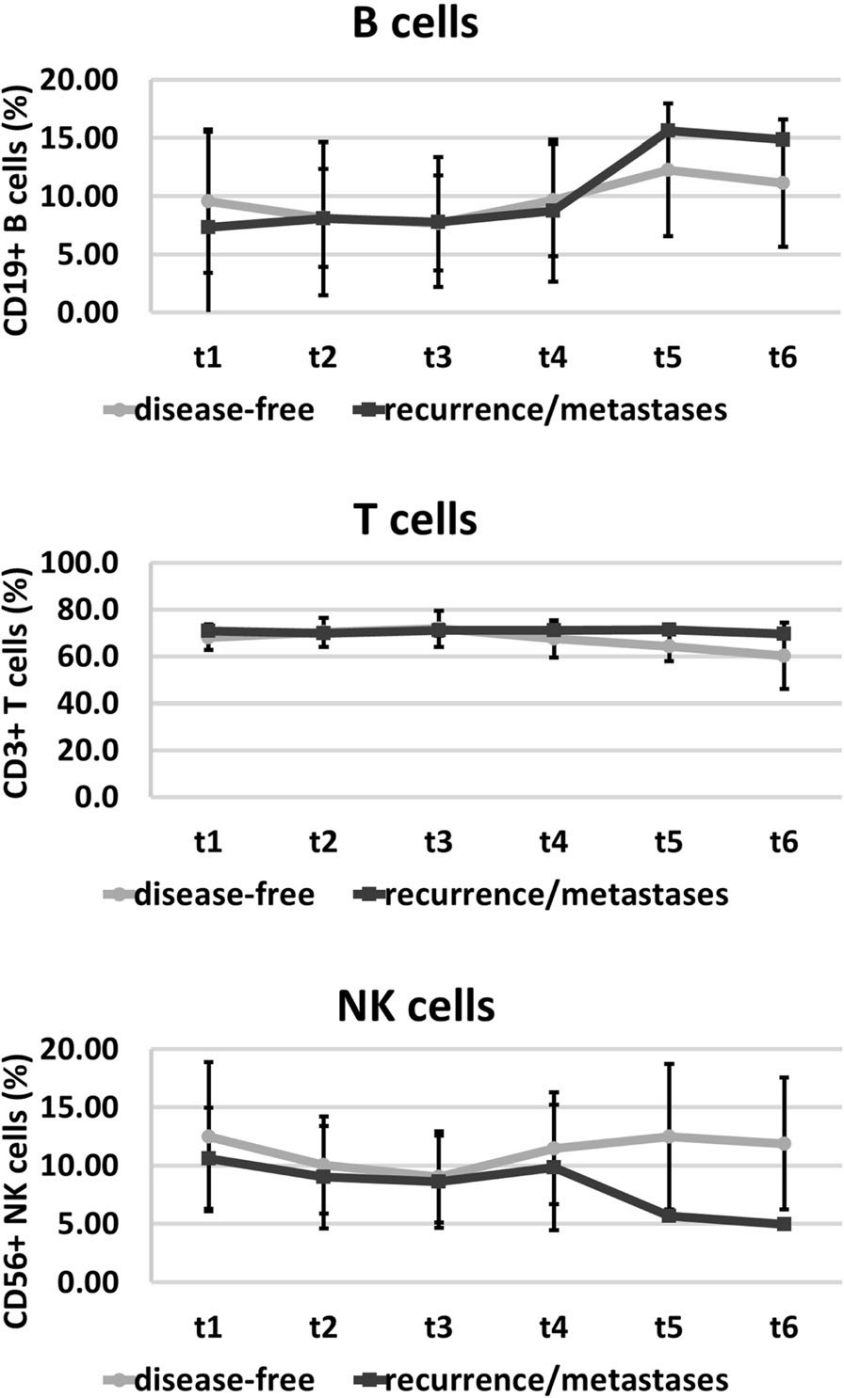
Immunophenotyping of the percentages of major lymphocyte subpopulations such as CD3^−^/CD56^+^ NK cells, CD3^+^ T cells, CD19^+^ B cells before (t1), after 30 Gy (t2), at the end of RT (t3), and in the follow-up period (t4, 6 weeks after RT; t5, 6 months after RT; t6, 1 year after RT) in disease-free patients (closed circles) and patients who developed recurrence or distant metastases (closed squares). The data show mean values ± standard deviation of the percentage of positively stained cells

The analysis of lymphocyte subpopulations in the peripheral blood revealed a drop in the percentage of NK cells 6 and 12 months after RT in recurrence/metastasize patients compared to disease-free patients, whereas the percentage of T and B cells remained nearly unaltered in the follow-up period (Fig. 3). The absolute numbers of NK cells in patients with contralateral recurrence/metastases remained always below that of disease-free patients throughout the whole course of disease, however, due to the low number of patients the data failed to show statistical significance (Table 7).

**Table 7:** Absolute NK cell counts (Giga (10^9^) per liter) during the course of disease in disease-free and patients with recurrence/metastasis (mean ± standard deviation)

	N	Before RT	After 30 Gy	End of RT	6 weeks after RT	6 months after RT	1 year after RT
		NK cell counts					
Disease-free	32	0.15 ± 0.08	0.12 ± 0.06	0.09 ± 0.05	0.14 ± 0.06	0.16 ± 0.09	0.18 ± 0.1
Recurrence/metastases	2	0.14 ± 0.03	0.07 ± 0.01	0.06 ± 0	0.11 ± 0	0.14 ± 0	0.11 ± 0

## Discussion

Two breast cancer patients out of 35 with elevated Hsp70 serum levels at the end of RT that appear to persist for 6 weeks after adjuvant RT developed contralateral recurrence or distant metastases 2 years after RT. Since the patients of this study were diagnosed as tumor-free after breast conserving surgery (R0 resection) and RT, it is assumed that the circulating, free Hsp70 which was measured in the serum by the R&D and lipHsp70 ELISA rather originates from chronically inflamed normal tissue than from dying tumor cells, which was shown in a lung cancer study, previously [13]. One might hypothesize that tumor cell regrowth might be promoted by chronic inflammation which is reflected by a long-lasting release of DAMPs, such as free Hsp70 for up to 6 weeks after RT. Chronic inflammation is known to promote tumor growth [23] via several mechanisms. Inflammatory mediators such as tumor necrosis factor-alpha (TNF-α), Interleukin-6 (IL-6), Interleukin-10 (IL-10) and transforming growth factor-beta (TGF-β) play important roles in tumorigenesis and the development of cancer in different entities [24]. In addition, reactive oxygen and nitrogen species are released in chronically inflamed tissues and thereby affect DNA integrity, which in turn might also increase the risk of cancer formation [25]. In an inflamed microenvironment, different DAMPs, which are normally localised in the cytosol, are released into the extracellular milieu where they can activate the immune cells, stimulate tissue repair mechanisms and thereby might mediate protective antitumor immunity [6, 10]. However, a chronic, long-term exposure of immune cells to DAMPs can induce immune-tolerance which promotes tumor growth and thereby enhances the risk of relapse or distant metastases [26]. Free Hsp70 as a representative for DAMPs was found to play an important role in tumor formation of different cancer entities, such as bladder cancer [27], colorectal carcinoma [28, 29], osteosarcoma [28], fibrosarcoma [30], melanoma [31] and T cell leukemia [32]. In the present study, free Hsp70 levels were elevated for at least 6 weeks after RT in two patients who showed recurrence or developed distant metastases. In contrast, a patient with secondary endometrial carcinoma had Hsp70 levels in the range of healthy individuals throughout the whole course of disease. This finding might be indicative for lower Hsp70 levels in this specific cancer type.

The function of intracellular Hsp70 as a molecular chaperone plays a major role in the correct folding of nascent proteins, and its antiapoptotic activity protects cancer cells against environmental stress [19]. Elevated extracellular Hsp70 levels fulfil dual roles, on the one hand they can stimulate antitumor immune responses, on the other hand, in the case of chronic exposure they might induce immune-tolerance. We hypothesize, that apart from Hsp70 also other DAMPs could be released into the circulation that further propagate chronic inflammation which in turn suppress antitumor immune responses and promote tumor growth. The patients with a long-term disease-free survival exhibited significantly lower free Hsp70 values in the serum that might be indicative for a faster clearance of the inflammatory response after RT.

Apart from free also exosomal Hsp70 which is actively released from viable tumor cells [14, 33] has been found at elevated levels in tumor patients. Since blood can be taken repeatedly by minimal invasive methods, the course of Hsp70 (free and exosomal) levels during therapy can be easily determined. Therefore, we speculate that measuring free as well as exHsp70 concentrations in the serum of tumor patients before, during and after therapy might provide predictive value with respect to determine chronic inflammation and viable tumor mass. These data might allow a more rapid and earlier therapy adaptation in patients with high-risk tumors. Since the percentage of T and B cells remained nearly unaltered, but that of NK cells dropped significantly in the follow-up period of patients with unfavourable prognosis, we speculate that NK cells might be important players in tumor control. In line with these findings we could show previously that a low NK cell infiltration in squamous cell carcinoma of the head and neck (SCCHN, *N* = 145) and a high Hsp70 expression are predictive for an adverse clinical outcome [34]. With respect to the absolute numbers, NK cell counts remained below that of disease-free patients throughout the whole course of disease in recurrence/metastases patients which might be due to the radiation therapy as shown by others [35–37]. The presence of extracellular Hsp70 derived from dying tumor cells in a pro-inflammatory environment provides a stimulatory signal for NK cell activation, as shown by in vitro studies [38, 39].

However, due to the low number of breast cancer patients who developed recurrence or distant metastases in our clinical trial further studies with larger patient cohorts are necessary to validate the value of Hsp70 serum levels and NK cell counts in the peripheral blood as predictors of clinical responses.

## Conclusions

Two breast cancer patients who developed contralateral recurrence or distant metastases showed longitudinal higher serum levels of free Hsp70 after RT than patients who remained disease-free. These findings are in line with data showing an unfavourable clinical outcome in SCCHN patients with a low NK cell infiltration and a high Hsp70 expression. Furthermore, NK cell counts in patients with adverse prognosis were always below that of disease-free patients. This finding might be explained by an irradiation-induced NK cell killing. In case that these findings could be confirmed in a larger patient cohort, longitudinal elevated levels of free Hsp70 levels after RT together with a drop in NK cell counts might be considered as a predictive marker for an unfavourable prognosis.
